# Enhancing Antimicrobial Efficacy of Sandalwood Essential Oil Against *Salmonella enterica* for Food Preservation

**DOI:** 10.3390/foods13233919

**Published:** 2024-12-04

**Authors:** Andrea Verešová, Margarita Terentjeva, Zhaojun Ban, Li Li, Milena Vukic, Nenad Vukovic, Maciej Ireneusz Kluz, Rania Ben Sad, Anis Ben Hsouna, Alessandro Bianchi, Ján Kollár, Joel Horacio Elizondo-Luévano, Natália Čmiková, Stefania Garzoli, Miroslava Kačániová

**Affiliations:** 1 Institute of Horticulture, Faculty of Horticulture and Landscape Engineering, Slovak University of Agriculture, Tr. A. Hlinku 2, 94976 Nitra, Slovakia; andrea.veresova1979@gmail.com (A.V.); n.cmikova@gmail.com (N.Č.); 2 Faculty of Veterinary Medicine, Latvia University of Life Sciences and Technologies, LV-3001 Jelgava, Latvia; margarita.terentjeva@llu.lv; 3 School of Biological and Chemical Engineering, Zhejiang University of Science and Technology, Zhejiang Provincial Key Laboratory of Chemical and Biological Processing Technology of Farm Products, Zhejiang Provincial Collaborative Innovation Center of Agricultural Biological Resources Biochemical Manufacturing, Hangzhou 310023, China; banzhaojun@zust.edu.cn; 4 Key Laboratory for Agro-Products Postharvest Handling of Ministry of Agriculture and Rural Affairs, College of Biosystems Engineering and Food Science, Zhejiang University, Hangzhou 310058, China; lili1984@zju.edu.cn; 5 Department of Chemistry, Faculty of Science, University of Kragujevac, 34000 Kragujevac, Serbia; milena.vukic@pmf.kg.ac.rs (M.V.); nvchem@yahoo.com (N.V.); 6 School of Medical & Health Sciences, University of Economics and Human Sciences in Warsaw, Okopowa 59, 01 043 Warszawa, Poland; m.kluz@vizja.pl; 7 Laboratory of Biotechnology and Plant Improvement, Centre of Biotechnology of Sfax, P.O. Box 1177, Sfax 3018, Tunisia; raniabensaad@gmail.com (R.B.S.); benhsounanis@gmail.com (A.B.H.); 8 Department of Environmental Sciences and Nutrition, Higher Institute of Applied Sciences and Technology of Mahdia, University of Monastir, Monastir 5000, Tunisia; 9 Department of Agriculture, Food and Environment, University of Pisa, Via del Borghetto 80, 56124 Pisa, Italy; alessandro.bianchi@phd.unipi.it; 10 Institute of Landscape Architecture, Faculty of Horticulture and Landscape Engineering, Slovak University of Agriculture, Tulipánová 7, 94976 Nitra, Slovakia; jan.kollar@uniag.sk; 11 Department of Chemistry, Faculty of Biological Sciences, Universidad Autónoma de Nuevo León, San Nicolás de los Garza 64455, Nuevo León, Mexico; joel.elizondolv@uanl.edu.mx; 12 Department of Chemistry and Technologies of Drug, Sapienza University, P. le Aldo Moro, 5, 00185 Rome, Italy; stefania.garzoli@uniroma1.it

**Keywords:** antimicrobial effect, antibiofilm activity, insecticidal activity, sandalwood essential oil, carrot model, anti-salmonella activity, shelf-life storage

## Abstract

The growing emphasis on food safety and healthier lifestyles, driven by industrial expansion and scientific priorities, has highlighted the necessity of managing harmful microorganisms to guarantee food quality. A significant challenge in this domain is the control of pathogens that are capable of forming biofilms, entering a sessile state that enhances their resistance to broad-spectrum antibiotics. Essential oils, renowned for their antibacterial properties, present a promising natural alternative for food preservation. In this study, we analyzed the chemical composition of *Santalum album* essential oil (SAEO) using GC-MS, identifying (Z)-α-santalol (57.1%) as the primary constituent. Antimicrobial activity was confirmed through disc diffusion and minimum inhibitory concentration (MIC) assays against Gram-positive and Gram-negative bacteria and yeast from the genus *Candida*. Additionally, *in situ* experiments demonstrated that vapor-phase SAEO effectively inhibited *Serratia marcescens* on the food model, supporting its potential as a natural preservative. MBIC assays, crystal violet staining, and MALDI-TOF MS analysis on *S. enterica* biofilms were used to further evaluate the antibiofilm effects of SAEO. The crystal violet assay revealed a strong antibiofilm effect, while the MALDI-TOF MS analysis showed changes in the bacterial protein profiles on both glass and plastic surfaces. SAEO also showed significant anti-*Salmonella* activity on vacuum-packed carrot slices. SAEO outperformed the control samples. The insecticidal activity against *Megabruchidius dorsalis* was also studied in this work, and the best insecticidal activity was found at the highest concentrations. These findings indicate that SAEO could serve as a valuable component in food preservation, with notable antibacterial and antibiofilm benefits.

## 1. Introduction

*Santalum album,* a member of the Santalaceae family, is widely referred to as white or East Indian sandalwood and represents the inaugural species of sandalwood to be formally identified. Other species within the *Santalum* genus are also regarded as true sandalwoods, distinguished from other plants that produce similarly scented wood or oil [1]. Sandalwood essential oil is one of the most valuable and widely used essential oils. It is extracted from trees of the Santalum album species and extensively applied in aromatherapy, cosmetics, and perfumes [2]. The popularity of naturally derived compounds stems from their ability to influence various signaling pathways involved in disease development, along with their generally safe profile. Historically, sandalwood essential oil has been utilized in the therapeutic management of various ailments, encompassing fever, colds, burns, headaches, urinary tract infections, and bronchitis [2]. In addition to its medical and industrial uses, sandalwood is culturally significant and part of traditional practices in many Asian households [3]. Due to its high market value, researchers are continually working to improve both the quality and yield of essential oil produced from *S. album* to meet global demand. In gastronomic contexts, sandalwood oil is conventionally employed at concentrations not exceeding 0.001% (10 ppm) as a flavoring agent in various products, including both alcoholic and non-alcoholic beverages, frozen dairy confections, confectionery items, baked goods, gelatinous desserts, and puddings. The maximum permissible concentration of sandalwood oil in food is approximately 90 parts per million, whether used alone or in conjunction with other natural flavoring substances [4].

*Salmonella* is primarily transmitted through direct contact with infected people or animals or through consuming contaminated food or water sources [5]. Common infections linked to *Salmonella* species include gastroenteritis, bacteremia, asymptomatic chronic carriage, and localized infections [6]. Research has shown that various species of *Salmonella* genera can form biofilms, which they achieve through diverse chemical cell signaling processes [7]. Biofilms consist of microbial communities with cells that attach to surfaces and are embedded within a matrix of self-produced polymers [8]. Research has demonstrated that *Salmonella* possesses the capability to form biofilms on abiotic surfaces such as plastic, rubber, cement, glass, and stainless steel [9]. This phenomenon has elicited a notable increase in scholarly interest regarding the discovery of innovative and effective antimicrobials aimed at addressing resistant bacterial strains. Owing to their varied chemical constituents, which encompass terpenes, phenols, and aldehydes, recent investigations have demonstrated that essential oils (EOs) exhibit considerable antibacterial efficacy [10,11,12]. This chemical diversity allows EOs to act against a range of microbes, including mechanisms such as the inhibition of quorum sensing (QS), prevention of biofilm formation, and reduction of resistant bacterial growth [13].

Microbial contamination can occur at various stages of the food processing chain, including through contact with soil, water, and during transportation and handling [14,15]. To extend the shelf life of food products, studies often focus on multiple aspects of quality, including microbiological safety, chemical and physical properties, and sensory attributes. For carrot-based foods, interventions targeting *Escherichia coli*, *Salmonella* Typhimurium, and *Listeria monocytogenes* are frequently documented [16]. Because carrots grow in and maintain direct contact with soil, they are at risk for *Salmonella* contamination. The risk is particularly elevated in ready-to-eat carrot slices produced by informal processing sectors [17]. According to Kamle et al. [18] and Pandey et al. [19], EOs and their constituents have antibacterial and food preservation qualities that protect vegetables, meats, fish, and fruits from a variety of diseases. When it comes to food safety, preservation, and packaging, EOs have amazing potential [20]. Salmonellosis ranks among the top causes of foodborne illnesses globally. Temperature regulation is a key factor influencing both the stability and survival of *Salmonella* spp., while other variables, such as the incorporation of plant essential oils, also impact these dynamics [21,22].

In addition to their antibacterial properties, plant essential oils have also been demonstrated to possess natural repellent effects. This has resulted in a growing interest in environmentally friendly, natural alternatives for pest control. The repellent efficacy of essential oils can be influenced by their volatility, with some plant-derived repellents demonstrating efficacy that is comparable to, or even superior to, that of synthetic alternatives. However, synthetic repellents typically exhibit greater potency and/or longer-lasting effects than their natural counterparts [23].

The primary goal of this research was to examine the chemical composition and significant biological activities of *Santalum album* essential oil (SAEO), encompassing its antimicrobial properties (both *in vitro* and *in situ*), its antibiofilm efficacy against *S. enterica*, and its insecticidal capabilities against *Megabruchidius dorsalis*. The secondary aim was to evaluate the shelf life of carrot slices subjected to various treatment methodologies, which included the direct application of *S. enterica* and the integration of sous vide techniques with *S. album* EO.

## 2. Materials and Methods

### 2.1. Santalum album Essential Oil

The essential oil (EO) employed in this investigation was procured through the steam distillation process of pulverized *Santalum album* wood (SAEO). This oil was acquired from Hanus s.r.o., located in Nitra, Slovakia, with its origin traced back to Australia. The EO was preserved at a temperature of 4 °C to ensure its integrity for subsequent analytical procedures.

### 2.2. Chemical analysis of SAEO Samples Using Gas Chromatography (GC) and Gas Chromatography–Mass Spectrometry (GC/MS)

The chemical constituents of *Santalum album* essential oil (SAEO) were meticulously examined by employing a quadrupole mass spectrometer (Agilent Technologies, Santa Clara, CA, USA) in conjunction with a 6890 N gas chromatograph. A semi-quantitative assessment of each recognized constituent was previously executed in the investigation conducted by Kačániová et al [24]. The discernment of individual volatile constituents was accomplished by utilizing two distinct methodologies. Initially, the mass spectral data were juxtaposed with reference spectra derived from the MS libraries (Wiley7 and NIST). To further substantiate and authenticate the findings, the retention indices (RIs) of the identified compounds were contrasted with those of a series of n-alkanes (C_7_–C_35_) [25,26]. The percentage composition of each component, exceeding 0.1%, was ascertained from the area of the corresponding GC peak.

### 2.3. Antimicrobial Testing

#### 2.3.1. Microorganisms

The antibacterial efficacy of *Santalum album* essential oil (SAEO) was evaluated against a spectrum of bacterial strains, encompassing Gram-negative strains such as *Salmonella enterica* subsp. *enterica* CCM 3807, *Serratia marcescens* CCM 8588, *Shigella sonnei* CCM 4421, and *Yersinia enterocolitica* CCM 7204T, in addition to Gram-positive bacteria including *Listeria ivanovii* CCM 5884, *Listeria monocytogenes* CCM 4699, *Streptococcus pneumoniae* CCM 451, and *Staphylococcus aureus* CCM 4423. Additionally, yeast strains such as *Candida albicans* CCM8136, *Candida glabrata* CCM827, *Candida krusei* CCM8271, *Candida parapsilosis* CCM 8260, and *Candida tropicalis* CCM8264 were included. All bacterial and yeast strains were procured from the Czech Collection of Microorganisms located in Brno, Czechia. For the assessment of antibiofilm activity, biofilm-forming *Salmonella enterica* isolated from milk production was utilized. The identification of the bacteria was conducted through molecular methodologies. The bacterial and yeast inocula were cultivated in Mueller–Hinton broth (MHB, Oxoid, Basingstoke, UK) and Sabouraud dextrose broth (SDB, Oxoid, Basingstoke, UK) for a duration of 24 h at 37 °C for bacteria and 25 °C for yeasts, respectively, prior to analysis. The optical density of the inocula was standardized to 0.5 McFarland standard on the day designated for the experiment [27].

#### 2.3.2. Disk Diffusion Method

A disc diffusion methodology was implemented for the determination of antimicrobial efficacy using the aforementioned microbial strains. Discs measuring 6 mm in diameter, infused with SAEO, were strategically placed on Mueller–Hinton agar (MHA, Oxoid, Basingstoke, UK) for bacterial cultures and on Sabouraud dextrose agar (SDA, Oxoid, Basingstoke, UK) for yeast cultures. The bacterial strains underwent incubation at 37 °C for a duration of 24 h, whereas the yeast strains were subjected to incubation at 25 °C. The diameters of the inhibition zones were recorded in millimeters. Control discs devoid of any treatment functioned as negative controls, while antibiotic and antifungal discs (cefoxitin for Gram-positive bacteria, gentamicin for Gram-negative bacteria, and fluconazole for yeasts, all sourced from Oxoid, Basingstoke, UK) were employed as positive controls [27].

#### 2.3.3. Minimum Inhibitory Concentration (MIC) Values

Minimum inhibitory concentrations were determined. A total of 50 µL of microbial inoculum was added to each well of a 96-well microplate. Various SAEO concentrations (10 mg/mL to 0.00488 mg/mL in MHB) were added [28]. Negative controls were MHB and SDB containing SAEO, while positive controls were SDB with inoculum. After incubation, absorbance was measured at 570 nm (Glomax, Promega Inc., Madison, WI, USA). MIC_50_ and MIC_90_ values were defined as the lowest concentrations of SAEO to inhibit 50% and 90% growth, respectively. The experiment was performed in triplicate [28].

### 2.4. In Situ Testing of Fruit and Vegetables

In order to gain a better understanding of the *in situ* antimicrobial activity of SAEO, a number of different substrates were tested, including commercial apple, pear, carrot, and potato, as well as specific yeast strains and both Gram-positive and Gram-negative bacteria [29]. The substrates were cut into 0.5 mm pieces, cleaned, and placed in 60 mm Petri dishes that had been inoculated with bacteria. SAEO was dispersed in ethyl acetate at concentrations of 500, 250, 125, and 62.5 μg/mL. Ethyl acetate filter sheets were used as controls. The plates were then sealed and incubated at 37 °C for seven days. Microbial colony growth was assessed using ImageJ to calculate bacterial and yeast volume densities, together with standard methods for measuring *in situ* colony development [28].

### 2.5. Antibiofilm Activity

#### 2.5.1. Assay with Crystal Violet

The bacterial suspensions were cultured in Mueller–Hinton broth at 37 °C, as previously described by Kačániová et al. [29], with the objective of evaluating the minimum concentration of SAEO that inhibits biofilm formation (MBIC). A series of dilutions of SAEO, ranging from 100 mg/mL to 0.049 mg/mL, were added to the wells of a microtiter plate containing the bacterial inoculum. Following a 24 h incubation period, the wells were cleaned, stained, and the absorbance was measured at 570 nm. The concentrations that resulted in a 50% and 90% reduction in biofilm formation were designated as MBIC_50_ and MBIC_90_.

#### 2.5.2. Detection of Biofilm Formation Using the MALDI-TOF MS Biotyper

The Bruker Daltonics MALDI-TOF MicroFlex instrument (Daltonics, Bremen, Germany) was employed for the assessment of protein degradation during biofilm formation. Polypropylene tubes containing 20 mL of Mueller–Hinton broth (MHB) and 100 μL of a *S. enterica* biofilm-forming inoculum were prepared with the use of plastic and glass slides, respectively. The experimental tubes were treated with SAEO at a concentration of 0.1%, while the control tubes were left untreated. The biofilms were collected from the plastic and glass surfaces through a three-day process of shaking the tubes at 170× *g*, followed by incubation at 37 °C over a period of 14 days. This procedure was then repeated at five, seven, nine, and twelve-day intervals. Furthermore, planktonic cells derived from the SAEO control samples were subjected to analysis. A dendrogram was constructed based on the Euclidean distance estimation method using 19 exemplary global spectra. Protein spectra were acquired through the utilization of MALDI-TOF (Daltonics, Bremen, Germany) in the linear positive ion mode as previously outlined [29].

### 2.6. Kinetic Growth

The growth curves for *S. enterica* were determined by measuring the optical density at 850 nm, using a personal bioreactor, the RTS-1, produced by Biosan, Riga, Latvia. The initial stage involved the cultivation of the bacterial strains on Mueller–Hinton agar (MHA; Oxoid, Basingstoke, UK) at 37 °C for a duration of 24 h. Subsequently, a single colony was transferred into a 30 mL sealed tube containing Mueller–Hinton broth (MHB) and incubated at 37 °C until the OD850 reached 1, indicating the onset of the logarithmic growth phase. The bioreactor was operated at 2000 rpm, with directional shifts occurring every second. The temperature was subsequently increased in increments over a period of 20 min to reach 50 °C, with optical density measurements taken at five-minute intervals. The procedure was then repeated at temperatures of 55 °C, 60 °C, and 65 °C. In a distinct experiment, a solution of 1% SAEO was introduced to the culture once the optical density at 850 nm had reached 1, indicating the commencement of the logarithmic phase. Optical density measurements were taken for the control sample, which did not include essential oil, and for the experimental sample, which did include essential oil. The bioreactor is calibrated to facilitate the cultivation of microorganisms with a typical size range of 0.4–0.8 × 1^−3^ μm [30].

### 2.7. Carrot Model of Sous Vide Antimicrobial Activity

The carrot samples (*Daucus carota*) used in this study, weighing a total of 480 g, were supplied by an authorized distributor in the Slovakia. The carrots were cut into 5 g pieces and transported to the microbiology laboratory. The experiment included three sets of raw carrot samples and two-hundred and forty treated and control samples each on day 1 and day 7. The carrot pieces were individually vacuum-sealed using a Concept vacuum packer. The treated samples were immersed in a 1% *v/w* solution of SAEO diluted in sunflower oil, while the control samples were both vacuum-sealed and left unpacked. A 100 µL inoculum of *S. enterica* subsp. *enterica* and 1% *v/w* SAEO solution was added to replicate the presence of the bacteria without causing harm to the carrot. Prior to vacuum packing, the samples were left to stimulate for about one minute [24].

The data that we have had access to during our evaluation are as follows:

(i) Control: New samples of carrots were stored in polyethylene bags (without vacuum) at a temperature of 4 °C. They were then treated at 50 to 65 °C for 5 to 20 min.
(ii) Control + vacuum: After being vacuum-packed in polyethylene bags and stored at 4 °C, fresh carrot samples were treated at 50–65 °C for 5–20 min.
(iii) EO: The collected carrot samples were vacuum packed, treated with 1% SAEO, and stored at 4 °C. They were then cooked at 50 to 65 °C for 5 to 20 min.
(iv) *Salmonella*: Fresh carrot samples, vacuum-packed and treated with *S. enterica*, were stored at 4 °C before exposure to the bacterium for 5–20 min at 50–65 °C.
(v) *Salmonella* + EO: Vacuum-packed fresh carrot samples treated with *S. enterica* and containing 1% SAEO were kept at 4 °C prior to being exposed to the bacterium at 50 to 65 °C for 5 to 20 min.

For the control, a raw carrot sample was used on the first day of the experiment. Following the application of SAEO on the first set of samples and *S. enterica* on the second, all samples were left to macerate for a day. Afterward, they were gently mixed and combined. The samples were then processed using a CASO SV1000 sous vide machine, produced by a company based in Arnsberg, Germany. Each sample group was prepared by cooking at a specific temperature for a set period, closely monitored throughout the sous vide cooking process.

The vacuum bags used in this study are made from high barrier polyethylene, a material known for its resistance to moisture and its ability to withstand extreme temperatures ranging from −30 °C to 100 °C. These pouches, with a thickness ranging from 40 to 200 microns, are also described as being free of bisphenol A, plasticizers, and microplastics. According to the product specifications, they are odorless and tasteless, durable, and suitable for long-term storage in freezers or chillers, maintaining their quality for several years.

### 2.8. Carrot Samples for Microbiological Analysis

A series of microbiological analyses were conducted over a 7-day period, commencing on day 0. Following heat treatment, the samples underwent evaluation on both day 1 and day 7. For each analysis, a 5 g quantity of carrot samples was measured and placed into a sterile stomacher bag. This was then diluted to a ratio of 1:10 with 45 mL of peptone water. The mixture was homogenized for two minutes using a stomacher. Afterward, 0.1 mL of the aliquot from the dilution was transferred onto standard pre-dried plate count agar (PCA, Oxoid, Basingstoke, UK). The samples were incubated for 30 min in a GFL 3031 rocking incubator (GFL, Burgwedel, Germany). Coliform bacteria were cultured on violet red bile lactose agar (VRBL, Oxoid, Basingstoke, UK) at 37 °C for 24–48 h, while total viable counts (TVCs) were plated on PCA and incubated at 30 °C for 48–72 h [24].

### 2.9. MALDI-TOF MS Biotyper Microbial Strain Identification

Using reference libraries and a matrix-assisted laser desorption/ionization time of flight (MALDI-TOF) with an MS Biotyper (Bruker, Daltonics, Bremen, Germany), microbial strains from sous vide carrot samples were identified. An organic material was made by mixing 50% acetonitrile, 47.5% water, and 2.5% trifluoroacetic acid to make a stock solution. A total of 500 µL of 100% acetonitrile, 475 µL of filtered water, and 25 µL of 10% trifluoroacetic acid were combined to create a 1 mL stock solution. The organic solvent was then combined with the “HCCA matrix portioned” in a 250 µL Eppendorf tube. In Vrable, Slovakia, Aloqence Science provided the matrix materials. The sample was prepared according to previously suggested guidelines [24].

### 2.10. Insecticidal Activity

The model organism, *Megabruchidius dorsalis* Fahreus, 1839, was used to evaluate the insecticidal activity of SAEO. Petri plates coated with sterile filter paper were used to hold groups of fifty *M. dorsalis* insects. In order to create different concentrations of SAEO, a 0.1% polysorbate solution (Aloquence, Vrable, Slovakia) was used to dilute the SAEO (100%, 50%, 25%, 12.5%, 6.25%, and 3.125%). Sterile filter paper discs were saturated with 100 µL of each SAEO concentration. The plates were then parafilm-sealed and allowed to sit at room temperature for a full day. One hundred microliters of the 0.1% polysorbate solution were given to the control group. The quantity of both living and dead insects was counted after a whole day. Three other research projects have successfully duplicated this experimental process [31].

### 2.11. Statistical Evaluation

Every measurement was carried out in triplicate, and the data are shown as mean values ± standard deviation (SD). Tukey’s HSD test was conducted with a significance threshold of *p* < 0.05 after a one-way ANOVA (CoStat version 6.451, CoHort Software, Pacific Grove, CA, USA). The JMP Pro 17.0 software (SAS Institute, Cary, NC, USA) was used for graphical presentations.

## 3. Results

### 3.1. Chemical Composition of the Essential Oil of Santalum album

Using the GC/MS method, the chemical makeup of SAEO’s volatile oil was examined. Table 1 presents the results, which indicate the percentage distribution of the volatile oil. A total of ten volatile compounds were found to account for 99.3% of the composition of the SAEO. The results suggest that the SAEO under investigation was primarily characterized by a significantly elevated percentage (57.1%) of (*Z*)-α-santalol, followed by a high abundance of (*Z*)-β-santalol (20.3%).

### 3.2. Disc Diffusion Antimicrobial Activity

Five yeast strains, four Gram-positive bacteria, four Gram-negative bacteria, and *S. enterica* biofilm-forming bacteria (BFB) were tested using the disc diffusion method to investigate the antimicrobial properties of SAEO. The findings showed that SAEO had the strongest antimicrobial activity against Gram-negative *Serratia marcescens* (13.33 mm), Gram-positive *Streptococcus pneumoniae* (20.33 mm), and also against yeast *Candida glabrata* and *C. parapsilosis* (8.33 mm).

Against the Gram-negative bacteria *S. enterica*, which forms a biofilm, the antibacterial activity of SAEO 15.33 mm was demonstrated. Compared to SAEO, the antibiotics have been shown to have greater antibacterial activity. *S. marcescens*, *Staphylococcus aureus*, and *C. albicans* showed the highest resistance to cefoxitin, gentamicin, and fluconazole (Table 2).

### 3.3. Broth Microdilution Method

The broth microdilution method was implemented to determine the minimal inhibitory concentrations, or MIC_50_ and MIC_90_. This was carried out in an effort to better understand SAEO’s antibacterial activity. Overall, SAEO was particularly successful at controlling Gram-positive organisms. The lowest MIC_50_ (0.122 mg/mL) and MIC_90_ values (0.144 mg/mL) were highlighted for *Streptococcus pneumoniae*. The MIC_50_ and MIC_90_ for Gram-negative *Serratia marcescens* and *Yersinia enterocolitica* were found to be 0.142 mg/mL, corresponding to 0.167 and 0.154 mg/mL, respectively. When *Salmonella enterica* was used, the formation of the biofilm was inhibited with SAEO, and the findings showed MIBC_50_ values of 0.148 mg/mL and MIBC_90_ values of 0.170 mg/mL. Using MIC_50_ and MIC_90_ levels of 0.174 and 0.190 mg/mL and 0.181 and 0.199 mg/mL, correspondingly, the yeast strains *Candida albicans* and *Candida krusei* demonstrated the best MIC outcomes. Table 3 provides the precise results of the minimum inhibitory concentration (MIC) assay.

### 3.4. In Situ Antimicrobial Activity on the Fruits and Vegetables

**Figure 1:**
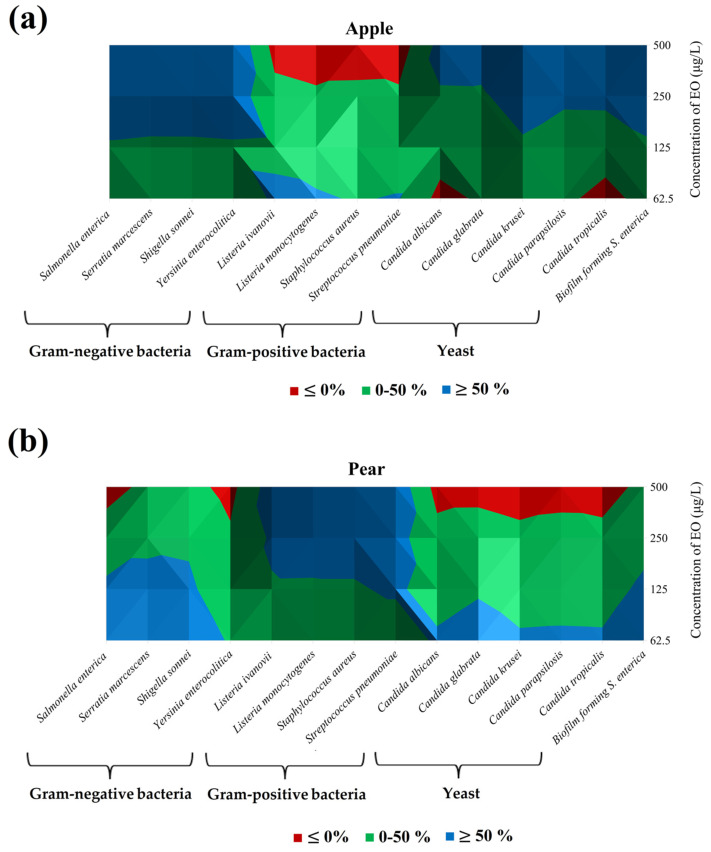
Isometric representation of the data from Table 4: (**a**) Apple; (**b**) Pear. Color scale: Red ≤ 0%; Green: 0–50%; Blue: ≥ 50%.

An *in situ* antimicrobial assay utilizing fruits and vegetables as food models was conducted. This experiment also incorporated an in vitro evaluation with selected bacteria. The vapor phase data for the fruit model are presented in Table 4 and Figure 1a,b. Overall, the SAEO vapor phase demonstrated greater efficacy in suppressing Gram-negative strains compared to yeast and Gram-positive bacteria. In the apple model, the highest concentration of SAEO (500 μg/L) achieved the significant inhibition of *S. marcescens* (94.08%). The maximum doses of SAEO effectively inhibited *S. enterica* and *Y. enterocolitica* (93.78% and 93.74%, respectively). SAEO inhibited biofilm-forming *S. enterica* (86.37%) at 500 μg/L and exhibited significant inhibition against *C. krusei* (87.55%). The strongest activity against *L. ivanovii* was recorded at 62.5 μg/L (55.75%). In the pear model, SAEO displayed the moderate inhibition of Gram-negative bacteria, with the highest impact on biofilm-forming *S. enterica* (78.84%) at 62.5 μg/L. In contrast, it inhibited *S. aureus* most effectively (86.95%) among Gram-positive strains at the highest concentrations. Additionally, SAEO effectively inhibited *C. glabrata* (67.58%) and *C. albicans* (56.33%) in yeasts at lower concentrations. However, at the lowest concentrations, some Gram-negative bacteria and yeast species showed increased growth, indicating the potential promotion of microbial growth by lower EO concentrations.

**Figure 2:**
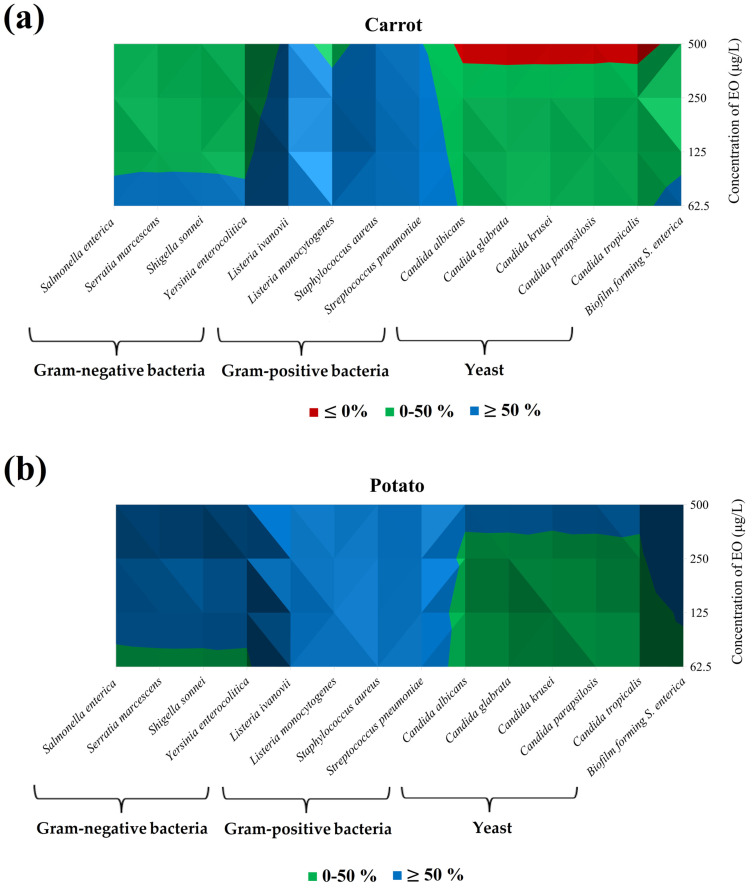
Isometric representation of the data from Table 5: (**a**) Carrot; (**b**) Potato. Color scale: Red ≤ 0%; Green: 0–50%; Blue: ≥50%.

In this phase of the experiment (Table 5, Figure 2a,b), we assessed the antimicrobial activity of SAEO on vegetables against various microorganisms in the vapor phase. The carrot model showed the highest inhibition against *S. pneumoniae* (87.39%) at 62.5 μg/L, followed by *S. aureus* (86.26%) and *L. ivanovii* (86.21%). In the potato model, the same 62.5 μg/L concentration effectively inhibited Gram-positive bacteria, while the highest concentration (500 μg/L) was most effective against Gram-negative bacteria, particularly biofilm-forming *S. enterica* (77.44%). *L. monocytogenes* and *S. aureus* were inhibited by 87.32% at 62.5 μg/L. Conversely, yeasts exhibited the lowest antimicrobial activity in the potato model at this concentration.

In conclusion, as shown in Figure 1a,b, the two fruit models (kiwi and banana) used had different trends in function of the type of microorganisms and the concentration of essential oil used. In apples, by increasing the concentration of SAEO used, the percentage of inhibition tended to increase, but not for Gram-positive bacteria, where the opposite trend was observed. For Gram-negative bacteria, BFB *S. enterica*, and yeasts, concentrations of 250 μg/L and 500 μg/L lead to an inhibition greater than 50%; indeed, for Gram-positive bacteria, this inhibition value was only maintained with 62.5 μg/L of SAEO (Figure 1a).

In pear, on the other hand, the 50% inhibition was archived for Gram-negative bacteria, BFB *S. enterica,* and yeasts for concentrations of less than 125 μg/L and for Gram-positive bacteria at 250 and 500 μg/L (Figure 1b).

In the vegetal models (carrot and potato) in Figure 2a,b, the percentage of inhibition varies depending on the microorganisms and the model used.

In carrot, at a concentration of 62.5 μg/L, the highest inhibition was detected, especially against Gram-positive bacteria. On the contrary, against yeasts, the lowest effect was found, and at concentrations of 62.5 μg/L, the growth of the microorganism was favored (Figure 2a).

In potato, the application of SAEO showed a major effect. All Gram-positive bacteria, Gram-negative bacteria, and BFB *S. enterica* were inhibited at concentrations higher than 125 μg/L, but yeasts were inhibited only at concentration of 500 μg/L (Figure 2b).

### 3.5. Antibiofilm Activity of Santalum album EO

**Figure 3:**
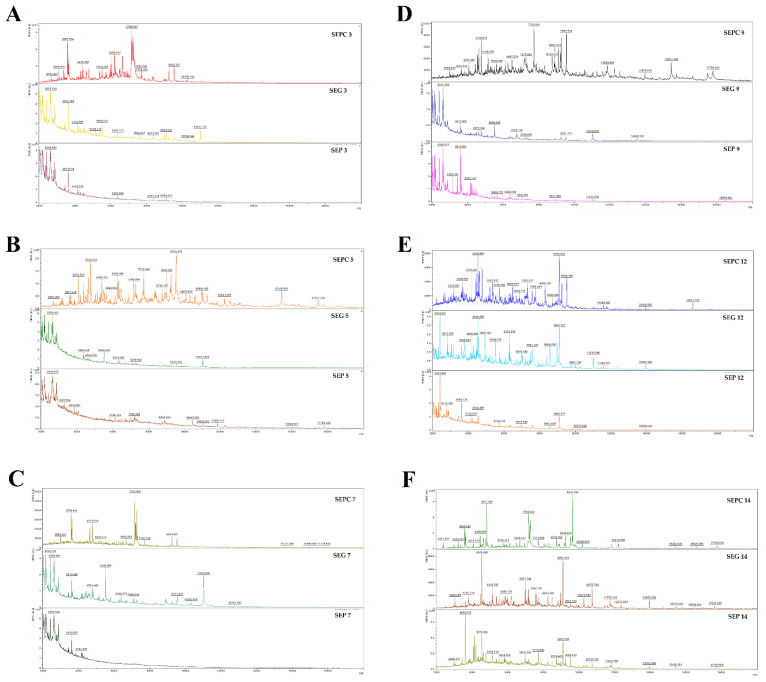
Representative MALDI-TOF mass spectra of *S. enterica*: (**A**) 3rd day, (**B**) 5th day, (**C**) 7th day, (**D**) 9th day, (**E**) 12th day, and (**F**) 14th day. SE = *S. enterica*; G = glass; P = plastic; PC = planktonic cells.

Individually or collectively, *S. enterica* can endure and develop greater resistance to antibiotic medicines as a result of biofilm development. SAEO exhibits antibiofilm activity against *S. enterica*, according to the crystal violet technique, with an MBIC_50_ of 0.148 mg/mL and an MIC_90_ of 0.170 mg/mL (Table 3). The antibiofilm effect of SAEO on glass and plastic surfaces against *S. enterica* was evaluated by mass spectrometry using the MALDI-TOF MS Biotyper. The spectra of untreated biofilm and planktonic cells, used as control samples, showed the same progression. As a result, the spectra of the planktonic cells served as the representative spectra of the control group. The experimental groups supplemented with SAEO were compared on both glass and plastic surfaces. In particular, at the beginning of the experiment (on the third day), there were noticeable differences between the experimental groups and the planktonic control spectrum. Both the number of peaks and the shape of the mass spectrum showed these variations. In contrast to the experimental spectrum from the glass surface, the spectrum from the plastic surface had fewer peaks (Figure 3A). As the experiment went on to day 5, the differences remained noticeable when compared to the control plankton spectrum, which had comparable peak shapes and numbers (Figure 3B). With the exception of day 14 (Figure 3F), when the spectra displayed a significant degree of uniformity, this pattern of differences continued throughout the trial (Figure 3C–E). The observed discrepancies between the experimental groups and the control spectra suggest that the application of SAEO led to the destruction and inhibition of the *S. enterica* biofilm. According to these findings, SAEO may be able to interfere with biofilm homeostasis early on. This could be a viable substitute to manage the development of *S. enterica* biofilms.

**Figure 4:**
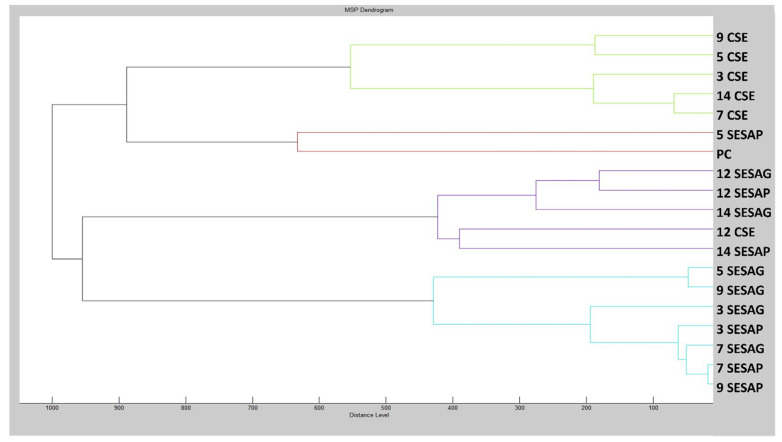
Dendrogram of *S. enterica* generated using MSPs of the planktonic cells and the control. SE = *S. enterica*; G = glass; P = plastic; PC = planktonic cells.

To demonstrate the parallels in biofilm structure between the control and experimental groups, a dendrogram was generated using the MSP (mass spectra) distances (Figure 4). In particular, the shortest MSP distances were observed in the early stages of the experimental biofilm groups on days 7 and 9 (7,9 SESAP) in conjunction with the control groups (7,9 CSE). As the duration of exposure of the experimental groups to the SAEO increased, the distances between the MSPs also increased. The most significant difference, particularly for the experimental group on the plastic surface (5 SESAP) and the control placental cells (PCs), was observed on the fifth day of the experiment, when the MSP distance of the experimental group was maximal. On the basis of these observations, it can be concluded that the SAEO has an effect on the homeostasis of the *S. enterica* biofilm and contributes to its inhibition. These findings are in agreement with the results of the analysis of the mass spectra.

### 3.6. S. enterica Growth Rate

**Figure 5:**
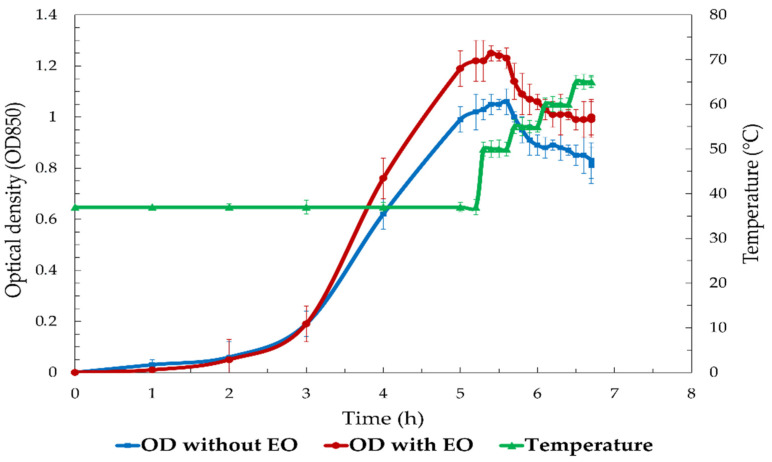
Optical density (OD850) of *S. enterica* over time with and without SAEO treatment at varying temperatures.

Figure 5 shows the growth of *S. enterica* over time, comparing the optical density (OD850) under two conditions: with 1% SAEO added at OD 1 (log phase) and no EO (control), alongside the temperature profile of an assay. *S. enterica* showed continuous growth during the first 3 h, reaching a peak OD of approximately 1.0 in the control (no EO) group. Bacterial growth stabilized after 3 h, followed by a gradual decrease in OD, indicating inhibited growth at higher temperatures, as the temperature increased above 40 °C. Conversely, the incorporation of 1% SAEO during the logarithmic phase yielded comparable growth patterns up to three hours, with an OD peak approaching 1.0. Following a three-hour incubation period, during which the temperature exceeded 40 °C, a notable decline in optical density was observed in the SAEO-treated group. The decline in optical density was more pronounced in the SAEO-treated group relative to the control group, suggesting that the presence of SAEO augmented the inhibitory impact of elevated temperatures on bacterial growth. At the end of the experiment (4.5 h), the OD in the SAEO-treated group had fallen to a significantly lower level than that in the control group, indicating a marked reduction in bacterial viability. These results highlight the combined effect of temperature and SAEO on the inhibition of bacterial growth.

**Figure 6:**
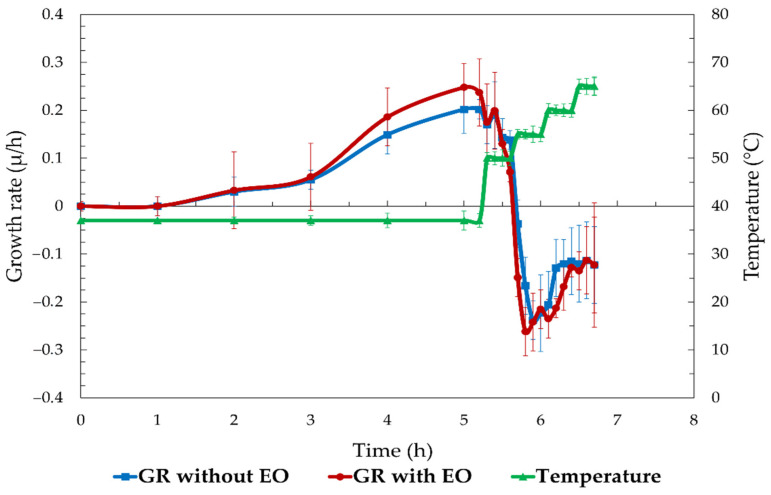
Growth rate (μ) of *S. enterica* over time with and without SAEO treatment at increasing temperatures.

Combined with the accompanying temperature profile, Figure 6 displays the growth rate (µ/h) of *S. enterica* over time under two experimental conditions: 1% SAEO inserted at the log phase (OD 1) and without EO (control). The bacterial development rate developed gradually in the control condition (no EO) until about 3 h, at which point it eventually reached a peak after about 2 h. Following this, as the temperature increased over 40 °C, the growth rate began to decrease. The growth rate exhibited a decline towards the conclusion of the 4.5 h interval, indicating that the elevated temperature had exerted an inhibitory effect on the bacteria. The rate of expansion in the SAEO-treated conditions exhibited a comparable pattern for the initial three hours of treatment, reaching a peak at approximately four hours, which was slightly higher than that observed in the control group. However, as the temperature kept on increasing after three hours had passed, the growth rate dramatically decreased and fell below the control. This dramatic drop in the development rate points to SAEO’s significant inhibitory effect, particularly at temperatures above 40 °C. The rate of proliferation in the SAEO-treated group was more negative at the end of the testing period than in the control group, demonstrating a deeper repression of bacterial growth.

The results presented here demonstrate that EO substantially decreases *S. enterica* development when combined with an increased temperature.

### 3.7. Sous Vide Carrot Microbiological Quality

**Figure 7:**
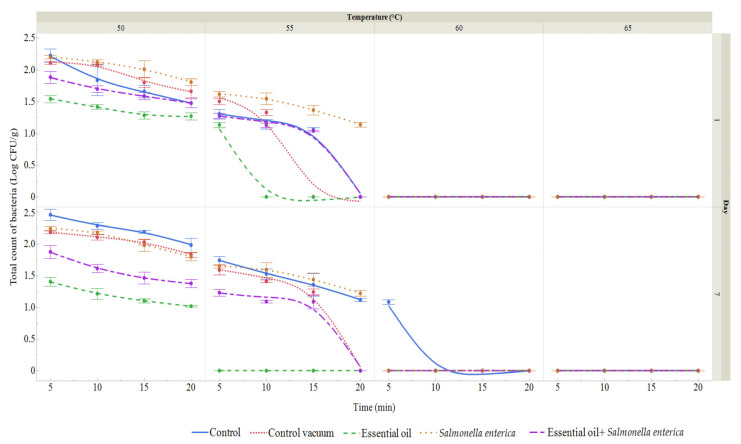
Total bacterial count (log CFU/g) in vacuum-packed carrots after heat treatment (50–65 °C, 5–20 min) on days 1 and 7. Data represent means ± SD (*n* = 3). Groups: Control (no treatment), Control Vacuum (vacuum-packed only), Essential Oil (1% SAEO), *S. enterica* (inoculated), and *S. enterica* + SAEO.

**Figure 8:**
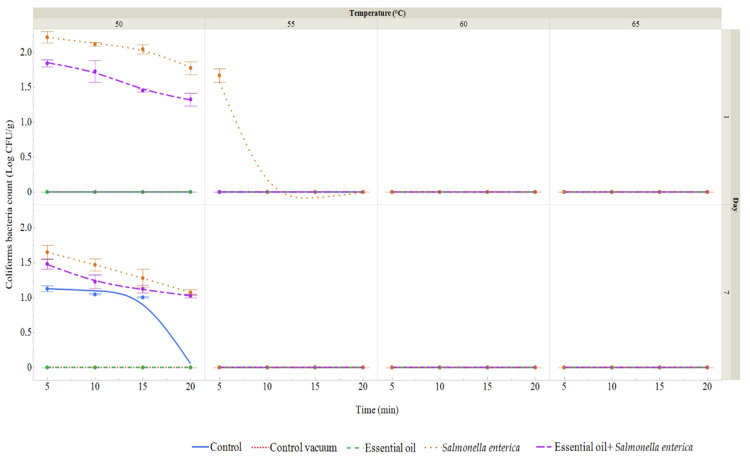
Coliform bacteria count (log CFU/g) in vacuum-packed carrots after heat treatment (50–65 °C, 5–20 min) on days 1 and 7. Data represent means ± SD (*n* = 3). Groups: Control (no treatment), Control Vacuum (vacuum-packed only), Essential Oil (1% SAEO), *S. enterica* (inoculated), and *S. enterica* + SAEO.

Raw carrots were microbiologically assessed for *S. enterica* using XLD agar. On day 0, no coliform bacteria were found. The total bacterial count (TBC) was 1.24 log CFU/g. For vacuum-packed carrots, TBC was evaluated on days 1 and 7 (Figure 7 and Figure 8). In the control group, bacterial counts ranged from 1.06 to 2.23 log CFU/g by day 7, while the vacuum-packed group showed counts of 1.33 to 2.11 log CFU/g on day 1 and 1.24 to 2.18 log CFU/g on day 7. In the SAEO-treated group, counts were lower, ranging from 1.13 to 1.54 log CFU/g on day 1 and 1.02 to 1.40 log CFU/g on day 7. For the *S. enterica* group, TBC ranged from 1.14 to 2.20 log CFU/g on day 1 and 1.22 to 2.24 log CFU/g on day 7. In the presence of *S. enterica*, TBC ranged from 1.04 to 1.88 log CFU/g on day 1 and 1.08 to 1.87 log CFU/g on day 7. Overall, the SAEO and *S. enterica* treatment groups exhibited lower bacterial counts.

Coliform bacteria (CB) were not detected in the SAEO treatment group, the vacuum-packed control group, or the uninoculated control group on day 1. CB was only found in the groups inoculated with *S. enterica*, ranging from 1.67 to 2.22 log CFU/g in the *S. enterica* group and from 1.33 to 1.84 log CFU/g in the SAEO + *S. enterica* group. On day 7, CB was 1.00–1.13 log CFU/g in the control group, but not detected in the SAEO or vacuum-packed groups. In the *S. enterica* inoculated group, CB was 1.07–1.65 log CFU/g on day 7, while in the SAEO + *S. enterica* group, it was lower at 1.02–1.48 log CFU/g.

Overall, the SAEO treatment resulted in lower coliform counts compared to the S. *enterica*-inoculated groups.

**Figure 9:**
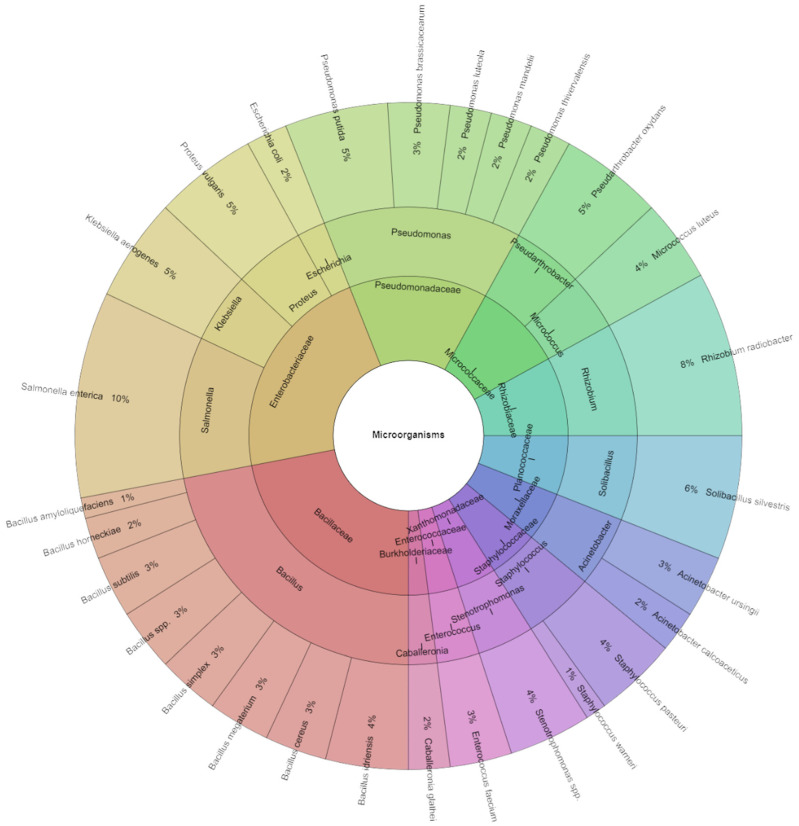
Krona diagram: species, genera, and families that were isolated from the sous vide carrot on the first day of storage.

Mass spectrometry identified 251 bacterial isolates from sous vide carrots (Figure 9), predominantly from the Bacillaceae and Pseudomonadaceae families. On day one, the most prevalent species were *Salmonella enterica* (10%), *Rhizobium radiobacter* (8%), and *Soliibacillus sylvestris* (6%).

**Figure 10:**
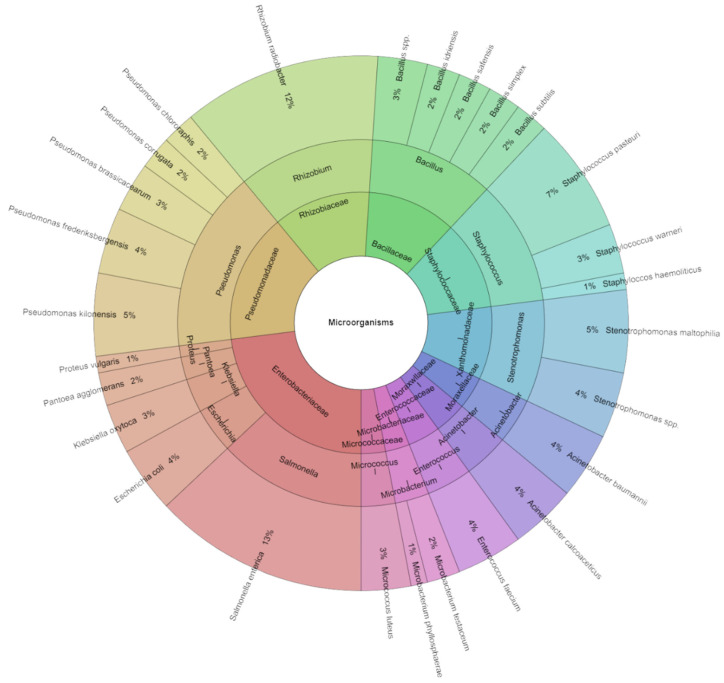
Krona diagram: species, genera, and families that were isolated from the sous vide carrot on the seventh day of storage.

The mass spectrometry analysis of sous vide carrots over seven days identified 234 bacterial isolates (Figure 10), primarily from the Pseudomonadaceae and Bacillaceae families. *S. enterica* (13%), intentionally inoculated, was the most prevalent species, followed by *Rhizobium radiobacter*, *Staphylococcus pasteuri*, *Pseudomonas kilonensis*, and *Stenotrophomonas maltophilia*.

### 3.8. SAEO Insecticidal Activity

Table 6 presents the insecticidal efficacy of SAEOs against *M. dorsalis*. The highest efficacy was observed at 50% and 100% concentrations of SAEO. At lower concentrations, no repellent effect was detected. However, a 12.5% concentration reduced the *M. dorsalis* population by 40%, while 25% concentration effectively killed 25% of the insects.

## 4. Discussion

Essential oils have long been used in many different ways, but in recent years, their use has significantly increased, especially in food, medicine, cosmetics, personal care items, and aromatherapy. Studies have increasingly highlighted the wide range of pharmacological benefits associated with EO components, including their antimicrobial, antioxidant, and soothing properties [32,33,34]. This study employed (GC-MS) to analyze the chemical composition of *Santalum album* essential oil (SAEO) obtained by steam distilling crushed wood. The results indicated that (Z)-α-santalol and (Z)-β-santalol were the dominant compounds, making up 77.4% of the oil’s composition. Additionally, the identified compounds in SAEO were categorized into four main classes: monoterpenes, sesquiterpenes, oxygenated terpenes, and other oxygenated substances [35]. Based on the literature data, and according to the results obtained herein, α-santalol and β-santalol, groups of oxygenated sesquiterpenes, are recognized as the volatile representatives of this oil. According to Lawrence [36], α-santalol and β-santalol, which make up around 8.7–25.2% and 7.1–48.6% of the EO produced from Indonesian sandalwood, respectively, are primarily responsible for the odor. Additionally, Nautiyal [37] found that 52.59% of the essential oil extracted from sandalwood is composed of santalol. In trade samples of *Santalum album* oil, the primary constituents, α- and β-santalol, account for 57–89% of the total composition [38]. These findings are further supported by a study that analyzed the chemical makeup of commercially available *S. album* essential oil [39]. Previous GC-MS analyses of sandalwood oils have reported similar chemical profiles [40,41,42,43]. To our knowledge, this study provides the most comprehensive comparison of the components present in oils from different *Santalum* species.

The best antimicrobial activity was found against Gram-negative bacteria in *S. marcescens*, against Gram-positive bacteria in *S. pneumoniae*, and against yeasts *C. glabrata* and *C. parapsilosis* using the disk diffusion method. SAEO was very effective in managing Gram-positive organisms with low concentrations of inhibition. *S. pneumoniae* was recognized as having the lowest concentration of inhibition. The bacteria most susceptible to SAEO were Gram-negative *Y. enterocolitica* and *S. marcescens*. *C. albicans* and *C. krusei* were the yeast strains that showed the best MIC results, according to the MIC. Jirovetz et al. [44] observed that the samples they tested exhibited antibacterial activity against both Gram-negative *Klebsiella pneumoniae* and Gram-positive *Staphylococcus aureus* in all trials. Sandalwood oils showed varying effects on Gram-negative bacteria such as *Escherichia coli* and *Pseudomonas aeruginosa*, while no activity was recorded against the yeast *Candida albicans* in the single *Santalum spicatum* sample examined via the agar diffusion method. Hammer et al. [45], using the agar dilution method, evaluated the in vitro activity of 24 essential oils, including sandalwood oil, against *Candida albicans* ATCC 10231. Additional studies have demonstrated that *Santalum album* oil exhibits activity against various Gram-positive bacterial strains, such as staphylococci—including those resistant to MRSA and VRSA—and streptococci, along with some Gram-negative bacteria [46]. SAEO has demonstrated antimicrobial activity against a diverse array of foodborne and human pathogens, as evidenced by previous studies [47,48]. Due to their low toxicity, economic viability, and biodegradability, essential oils may be a superior alternative to conventional antibiotics as a means of medication [49]. Nevertheless, the antimicrobial activity of essential oils does not exceed that of conventional antibiotics. In light of the aforementioned findings, it can be concluded that the antimicrobial efficacy of essential oils is on par with that of synthetic drugs, even when different concentrations of essential oils are taken into account. A comparison of the antimicrobial activity of essential oils with that of antibiotics is becoming an increasingly popular area of research in microbiology and medical science [50]. In contrast, antibiotics are either synthetic or semi-synthetic compounds that are specifically designed to target and kill or inhibit the growth of bacteria. However, this can result in the development of resistance over time, whereby the target of the antibiotic is altered or efflux pumps are developed to expel the drug [51]. The testing range for sandalwood oil was 0.03–2% (*v/v*). There was no discernible suppression of *Candida albicans* growth at 0.03% sandalwood oil. Sandalwood oil inhibited *Candida albicans* with the lowest MIC, at 0.06%, when compared to other oils. In an additional investigation, Morris et al. [52] examined the antimicrobial properties of several aroma components, such as sandalwood oil, against a diphtheroid, *C. albicans*, *Escherichia coli*, and *Staphylocccus aureus*. Sandalwood oil’s minimum inhibitory concentration for each of these four microbes was found to be 50, >1000, >1000, and 500 ppm, respectively. Additionally, another investigation found that sandalwood oil had the strongest inhibitory effects on *E. coli* and *Bacillus mycoides* [53]. Sandalwood oil showed no activity against *Candida albicans*, *Aspergillus niger*, and *Aspergillus fumigatus*, but it was effective against human pathogenic fungi such as *Microsporum canis*, *Trichophyton mentagrophytes*, and *Trichophyton rubrum* [54]. Furthermore, the compound epi-β-santalene exhibited activity against *Salmonella typhimurium*, while α- and β-santalol, components of sandalwood oil, demonstrated effectiveness against both *Staphylococcus aureus* and *S.* Typhimurium [55].

The crystal violet technique indicates that SAEO has antibiofilm activity against *S. enterica*, and mass spectrometry utilizing the MALDI-TOF MS Biotyper was used to assess SAEO’s antibiofilm effect on glass and plastic surfaces against *S. enterica*. The observed differences between the control and experimental groups’ spectra imply that the *S. enterica* biofilm was inhibited and destroyed as a result of SAEO administration. In the study by Kačániová et al. [28], the molecular profile of *Citrus limon’s* antibiofilm action against *S. enterica* was assessed. The earliest MSP distances were noted at the beginning of the experimental biofilm groups on day three, using dendrograms derived from MSP spectra. Microorganisms are resilient and have developed numerous defenses against environmental influences and antimicrobial therapies [56]. Therefore, in order to successfully develop novel antimicrobial drugs and address the antibiotic resistance dilemma, research and comprehension of their resistance mechanism are essential [57]. Encased in a matrix, biofilm-forming bacteria pose a significant medical risk because they defend themselves against the host’s immune system and drugs [58]. Therefore, the right choice of cleaning and sanitizing materials is important for the reduction in microbial adhesion. EOs can play an important role in preventing the growth of biofilms when added to sanitizing and cleaning solutions [59]. EO inhibited the production of biofilms, eliminated preformed *S. enteritidis* biofilms, and decreased biofilm biomass and biofilm cell activity in the Wang et al. [60] study.

In our study, we further explored the antimicrobial effects of essential oils (EOs) in the vapor phase using models of apple, pear, carrot, and potato, observing the most significant antimicrobial activity against *Serratia marcescens* on the fruit model and against biofilm-forming *Salmonella enterica* on the vegetable models. One approach to reducing the required concentration of EOs to achieve antimicrobial effects is utilizing their vapor phase, which has been suggested as an alternative for lowering the EO dose needed to inhibit or inactivate microorganisms [61]. This vapor phase bioactivity of EOs could serve as a promising method for preserving fresh produce during storage, showing encouraging results, particularly against bacteria and fungi [62,63,64]. However, it is noted that the antimicrobial activity of EOs in the vapor phase is often less potent in food systems with microbial cells compared to in vitro studies [65]. Numerous EOs, and the specific compounds within them, exhibit antibacterial properties against a broad spectrum of microbes. The antimicrobial effects in the vapor phase are attributed to the volatile components present in the EOs. Kačániová et al. [66] showed that the vapor phase of *Cedar atlantica* EO inhibited *Penicillium* fungi on celery, carrots, and bread. In an apple model infected with Gram-positive bacteria, *Pelargonium graveolens* EO proved most effective against *Enterococcus faecalis* at a concentration of 62.5 µg/mL. *PGEO* was the most efficient in inhibiting *Bacillus megaterium* at the same concentration in a carrot model [67]. Additionally, *Citrus limon* EO at 500 µg/mL was found to be most effective against *Staphylococcus aureus*, with concentrations of 62.5 µg/mL and 125 µg/mL showing effectiveness against *S. aureus* in an apple model. When testing the inhibitory effects on Gram-positive bacterial strains in the carrot model, *Citrus limon* EO was most potent against *S. aureus* at 500 µg/mL, while *Micrococcus luteus* and *Bacillus cereus* were suppressed only at the lowest oil concentration. The strongest vapor phase activity of *Citrus limon* EO against Gram-positive bacteria on kohlrabi was observed in an *in situ* study at 500 µg/mL, particularly against *S. aureus* [28]. Additional research under *in vivo* and *in situ* conditions is needed to thoroughly assess the full therapeutic potential of SAEO investigated, which could provide valuable insights into potential treatments as substitutes against relevant microorganisms.

Consistent with prior studies, *Salmonella enterica* demonstrated vigorous growth in the initial hours [68]. However, temperatures ranging from 60 to 65 °C for several minutes are generally effective in eliminating *Salmonella*, even when present in concentrations as high as one million per gram [69]. This highlights the effectiveness of thermal treatments in controlling this pathogen. A study by Guillín et al. [13] tested 15 different EOs for their antimicrobial and antibiofilm potential against *Salmonella enterica* growth. They confirmed that the EOs evaluated represent a promising alternative for microbial control and therapeutic treatment against pathogenic resistant bacteria. In our study, we combined elevated temperature with a 1% addition of *Santalum album* EO to further enhance the inhibitory effects on *Salmonella enterica*, which was also confirmed in our findings.

The subsequent phase of our investigation entailed examining the efficacy of *E. enterica* and SAEO in prolonging the shelf life of sous vide carrots. The results demonstrated that SAEO prolonged the shelf life of sliced carrots. In this experiment, the impact of varying temperatures and cooking methods on the shelf life of carrots was further examined. The results demonstrate that SAEO is an effective preservative. In addition to the enumeration of microorganisms, the species identified on carrots during storage were also documented. It is of paramount importance to conduct a thorough evaluation of the potential hazards associated with sous vide products, particularly those pertaining to spore-forming pathogens. Many pathogenic bacteria that cause foodborne illness have a maximum growth temperature range of 42 to 49 °C, although a few have been observed to grow more slowly at temperatures between 50 and 55 °C. Consequently, the temperatures employed in sous vide cooking may fall within or near the ranges where foodborne pathogens can develop [70]. In our study, *Rhizobium radiobacter* was the second most frequently isolated species after *S. enterica*. Vegetables are commonly contaminated by spoilage organisms such as *Escherichia coli*, *Enterobacter* spp., *Klebsiella* spp., *Salmonella* Typhi, *Serratia* spp., *Providencia* spp., *Staphylococcus aureus*, *Pseudomonas aeruginosa*, and other potentially pathogenic microorganisms [71]. Additionally, some vegetables are particularly prone to spoilage from other microorganisms, including *Bacillus cereus*, *Campylobacter jejuni*, *Clostridium botulinum*, *E. coli* O157, *Listeria monocytogenes*, *Salmonella* spp., *Shigella*, *Staphylococcus*, and *Vibrio cholera* [72]. Many of these organisms are facultative anaerobes, allowing them to survive and grow in both aerobic and anaerobic environments. A similar study conducted by Kačániová et al. [28] demonstrated the antimicrobial effects of *Citrus limon* EO against *S. enterica* on sous vide carrot models. In a study by Sebastiá et al. [73], various sous vide-cooked vegetables, including broccoli, courgette, potatoes, and carrots (cooked at 100 °C for 15–20 min, except for courgette which was heated for 5 min), were tested for microbiological quality after being cooled to below 3 °C and stored for 0, 15, and 30 days. During each storage period, broccoli exhibited the highest aerobic plate counts. The authors attributed this to the natural structure of broccoli, where hydrophobic pockets in the inflorescences can trap organic material not removed by washing. To improve disinfection, a chlorine treatment was recommended to reduce organic material levels and enhance the effectiveness of the cleaning process [74,75,76].

The findings of our study indicated that the maximum levels of insecticidal efficacy were observed when 50% and 100% of the tested SAEO were applied. Sandalwood oil has been used as an acaricide and repels the insect *Varroa jacobsoni* Oud. in honeybee colonies [77]. A moderate level of action was reported against the mushroom fly, *Lycoriella mali* [78]. Moreover, termites cannot penetrate the oil [79,80]. Furthermore, because of its acaricidal and oviposition-deterring properties, santalol has been demonstrated to be effective against the spider mite *Tetranychus urticae* [81,82].

## 5. Conclusions

Microbial foodborne diseases represent a considerable public health dilemma. Within this framework, a significant risk factor is associated with consumer preferences for “ready-to-eat” or minimally processed (MP) fruits and vegetables. The application of SAEO presents a feasible solution for reducing pathogenic bacterial presence and extending the shelf life of MP foods, in light of the health risks involved. Moreover, *in situ* investigations utilizing models of fruits and vegetables have validated the practical utility of SAEO in food preservation, underscoring its ability to inhibit microbial growth in vapor phase settings. The study also revealed noteworthy antibiofilm activity, demonstrating SAEO’s potential to counteract biofilm development, which remains a critical issue in public health and food safety. Furthermore, the results indicated that sous vide conditions could enhance the antimicrobial effectiveness of SAEO, thereby proposing a novel approach for the incorporation of essential oils into culinary methodologies to improve food safety. The investigations into the insecticidal activity further substantiated the function of essential oils (EOs) as natural deterrents against pests, highlighting their potential applicability in sustainable agricultural methodologies. In light of the escalating apprehensions regarding antibiotic resistance, SAEO emerges as a compelling candidate for additional inquiry as a safe and efficacious antimicrobial agent across diverse sectors. Subsequent research endeavors should concentrate on delineating the specific mechanisms underlying the action of SAEO’s constituents and assessing its efficacy within actual food systems and agricultural methodologies. To augment the stability and efficacy of SAEO, investigations could examine its formulation in conjunction with other natural preservatives and evaluate any potential synergistic effects when paired with conventional antibacterial agents. SAEO has the potential to catalyze innovative solutions to pressing issues in food safety and pest management.

## Figures and Tables

**Table 1:** Chemical composition of SAEO.

No	Compound ^a^	% ^b^	RI (lit.)	RI (calc.) ^c^
1	α-santalene	0.7	1417	1418
2	epi-β-santalene	1.9	1447	1448
3	β-santalene	2.8	1459	1460
4	*(Z)-*α-santalol	57.1	1675	1676
5	*(Z)*-α-trans-bergamotol	5.8	1690	1689
6	*(Z)*-epi-β-santalol	3.3	1702	1703
7	*(Z)*-β-santalol	20.3	1716	1716
8	*(Z)*-nuciferol	2.5	1725	1724
9	*(E)*-β-santalol	2.1	1739	1739
10	*(Z)*-lanceol	2.8	1761	1761
	total	99.3		

The literature values of retention indices on HP-5MS column; ^a^ identified compounds, ^b^ percentage amounts of identified compounds, ^c^ calculated values of retention indices on HP-5MS column.

**Table 2:** Antimicrobial activity of SAEO with disc diffusion method in mm.

Microorganism	Inhibition Zone	ATB
G-negative bacteria		
*Salmonella enterica* CCM 3807	11.33 ± 0.58 ^f^	30.67 ± 0.57 ^abc^
*Serratia marcescens* CCM 8588	13.33 ± 0.58 ^e^	31.33 ± 0.58 ^ab^
*Shigella sonnei* CCM 4421	12.67 ± 0.58 ^ef^	30.33 ± 0.59 ^abcd^
*Yersinia enterocolitica* CCM 7204T	11.33 ± 0.57 ^f^	30.33 ± 0.58 ^abcd^
G-positive bacteria		
*Listeria ivanovii* CCM 5884	16.33 ± 0.58 ^cd^	30.66 ± 0.57 ^abc^
*Listeria monocytogenes* CCM 4699	17.67 ± 0.59 ^bc^	31.33 ± 0.58 ^ab^
*Staphylococcus aureus* CCM 4423	18.67 ± 0.56 ^ab^	31.67 ± 0.57 ^a^
*Streptococcus pneumoniae* CCM 4501	20.33 ± 0.58 ^a^	31.34 ± 0.58 ^ab^
Yeast		
*Candida albicans* CCM 8186	7.67 ± 0.58 ^g^	29.67 ± 0.60 ^bcde^
*Candida glabrata* CCM 8270	8.33 ± 0.59 ^g^	28.33 ± 0.59 ^e^
*Candida krusei* CCM 8271	7.66 ± 0.58 ^g^	29.33 ± 0.58 ^cde^
*Candida parapsilosis* CCM 8260	8.33 ± 0.59 ^g^	28.67 ± 0.59 ^de^
*Candida tropicalis* CCM 8264	7.67 ± 0.57 ^g^	29.34 ± 0.58 ^cde^
Biofilm forming bacteria (BFB)		
*Salmonella enterica*	15.33 ± 0.58 ^d^	30.34 ± 0.58 ^abcd^

Data are the mean (±SD) of 3 samples. Different letters in each column refer to significant differences (Tukey, *p* ≤ 0.05). ATB = Antibiotics (Gram-positive bacteria, gentamicin for Gram-negative bacteria, and fluconazole for yeasts).

**Table 3:** Minimal inhibition concentration (MIC_50_ and MIC_90_) of SAEO in mg/mL.

Microorganism	MIC_50_	MIC_90_
G-negative bacteria		
*Salmonella enterica*	0.144 ± 0.011 ^abcd^	0.165 ± 0.009 ^bcd^
*Serratia marcescens*	0.142 ± 0.022 ^bcd^	0.167 ± 0.011 ^bcd^
*Shigella sonnei*	0.135 ± 0.010 ^cd^	0.159 ± 0.007 ^cde^
*Yersinia enterocolitica*	0.142 ± 0.012 ^bcd^	0.154 ± 0.012 ^cde^
G-positive bacteria		
*Listeria ivanovii*	0.128 ± 0.006 ^d^	0.148 ± 0.005 ^cde^
*Listeria monocytogenes*	0.124 ± 0.010 ^d^	0.134 ± 0.009 ^e^
*Staphylococcus aureus*	0.124 ± 0.010 ^d^	0.138 ± 0.006 ^de^
*Streptococcus pneumoniae*	0.122 ± 0.005 ^d^	0.144 ± 0.010 ^cde^
Yeast		
*Candida albicans*	0.174 ± 0.006 ^abc^	0.190 ± 0.006 ^ab^
*Candida glabrata*	0.188 ± 0.014 ^a^	0.197 ± 0.013 ^a^
*Candida krusei*	0.181 ± 0.032 ^ab^	0.199 ± 0.004 ^a^
*Candida parapsilosis*	0.184 ± 0.024 ^ab^	0.199 ± 0.009 ^a^
*Candida tropicalis*	0.184 ± 0.005 ^ab^	0.190 ± 0.016 ^ab^
Biofilm forming bacteria (BFB)		
*Salmonella enterica*	0.148 ± 0.016 ^abcd^	0.170 ± 0.015 ^abc^

Data are the mean (±SD) of 3 samples. Different letters in each column refer to significant differences (Tukey, *p* ≤ 0.05).

**Table 4:** *In situ* analysis of the antimicrobial activity (%) of the vapor phase of SAEO in fruit model.

Food Model	Microorganisms	Concentration of SAEO in μg/L			
		62.5	125	250	500
Apple					
G-negative bacteria	*Salmonella enterica*	23.23 ± 1.46 ^b^	46.03 ± 2.46 ^a^	74.55 ± 3.98 ^a^	93.78 ± 2.22 ^a^
	*Serratia marcescens*	26.02 ± 1.67 ^b^	43.44 ± 2.01 ^a^	73.75 ± 2.21 ^ab^	94.08 ± 3.90 ^a^
	*Shigella sonnei*	24.07 ± 2.62 ^b^	43.57 ± 1.99 ^a^	74.21 ± 3.31 ^ab^	93.45 ± 3.37 ^a^
	*Yersinia enterocolitica*	25.33 ± 4.21 ^b^	45.29 ± 3.56 ^a^	75.76 ± 3.93 ^a^	93.74 ± 2.28 ^a^
G-positive bacteria	*Listeria ivanovii*	55.75 ± 1.01 ^a^	43.86 ± 2.74 ^a^	14.00 ± 3.39 ^e^	−16.44 ± 1.63 ^ef^
	*Listeria monocytogenes*	53.82 ± 2.79 ^a^	34.22 ± 3.34 ^b^	6.63 ± 1.98 ^e^	−24.67 ± 2.91 ^fg^
	*Staphylococcus aureus*	47.66 ± 0.94 ^a^	23.89 ± 2.69 ^e^	7.02 ± 2.35 ^e^	−15.29 ± 3.37 ^e^
	*Streptococcus pneumoniae*	52.80 ± 6.03 ^a^	25.29 ± 3.00 ^de^	7.91 ± 1.17 ^e^	−26.28 ± 2.45 ^g^
Yeast	*Candida albicans*	−15.33 ± 2.73 ^d^	25.66 ± 2.21 ^cde^	45.60 ± 2.31 ^d^	66.05 ± 2.32 ^cd^
	*Candida glabrata*	6.97 ± 1.40 ^c^	23.89 ± 3.48 ^e^	46.13 ± 1.54 ^d^	63.40 ± 2.10 ^d^
	*Candida krusei*	25.70 ± 1.88 ^b^	45.00 ± 2.19 ^a^	64.85 ± 3.18 ^bc^	87.55 ± 2.80 ^a^
	*Candida parapsilosis*	14.02 ± 3.32 ^c^	32.66 ± 1.65 ^bcd^	56.03 ± 3.37 ^c^	75.91 ± 4.07 ^b^
	*Candida tropicalis*	−23.66 ± 2.80 ^d^	33.37 ± 1.45 ^bc^	56.39 ± 2.77 ^c^	74.85 ± 3.68 ^bc^
BFB	*Salmonella enterica*	24.65 ± 2.80 ^b^	46.54 ± 2.32 ^a^	63.10 ± 6.26 ^c^	86.37 ± 2.94 ^a^
Pear					
G-negative bacteria	*Salmonella enterica*	76.39 ± 3.54 ^a^	55.44 ± 2.40 ^a^	34.00 ± 1.25 ^e^	−25.96 ± 3.29 ^f^
	*Serratia marcescens*	75.03 ± 3.73 ^a^	55.15 ± 1.79 ^a^	46.50 ± 2.17 ^c^	15.70 ± 2.87 ^c^
	*Shigella sonnei*	76.26 ± 2.13 ^a^	56.97 ± 2.30 ^a^	44.08 ± 2.04 ^cd^	15.60 ± 3.19 ^c^
	*Yersinia enterocolitica*	45.95 ± 1.79 ^d^	23.19 ± 1.52 ^d^	7.55 ± 0.78 ^h^	−15.00 ± 2.68 ^d^
G-positive bacteria	*Listeria ivanovii*	24.74 ± 3.05 ^e^	46.33 ± 2.37 ^b^	56.33 ± 2.68 ^b^	74.89 ± 1.77 ^b^
	*Listeria monocytogenes*	25.30 ± 1.70 ^e^	45.96 ± 2.89 ^b^	64.16 ± 3.26 ^a^	85.16 ± 2.22 ^a^
	*Staphylococcus aureus*	24.96 ± 2.52 ^e^	45.96 ± 3.29 ^b^	66.24 ± 3.40 ^a^	86.95 ± 3.24 ^a^
	*Streptococcus pneumoniae*	24.60 ± 2.17 ^e^	56.26 ± 2.80 ^a^	66.74 ± 1.89 ^a^	85.16 ± 1.78 ^a^
Yeast	*Candida albicans*	56.33 ± 1.81 ^c^	33.26 ± 2.12 ^c^	14.92 ± 2.78 ^g^	−15.95 ± 3.13 ^de^
	*Candida glabrata*	67.58 ± 2.06 ^b^	45.95 ± 2.89 ^b^	23.15 ± 0.51 ^f^	−15.67 ± 1.92 ^d^
	*Candida krusei*	54.70 ± 2.23 ^c^	35.29 ± 3.16 ^c^	13.85 ± 2.54 ^gh^	−25.66 ± 1.92 ^f^
	*Candida parapsilosis*	55.81 ± 1.07 ^c^	35.70 ± 2.68 ^c^	16.62 ± 2.77 ^fg^	−16.86 ± 4.56 ^de^
	*Candida tropicalis*	55.21 ± 2.39 ^c^	34.60 ± 2.17 ^c^	16.03 ± 2.30 ^g^	−24.00 ± 2.55 ^ef^
BFB	*Salmonella enterica*	78.84 ± 0.95 ^a^	57.51 ± 0.64 ^a^	37.49 ± 2.54 ^de^	13.89 ± 1.60 ^c^

Data are the mean (±SD) of 3 samples. Different letters in each column (for each fruit model: apple and pear) refer to significant differences (Tukey, *p* ≤ 0.05).

**Table 5:** *In situ* analysis of the antimicrobial activity (%) of the vapor phase of SAEO in vegetable model.

Food Model	Microorganisms	Concentration of SAEO in μg/L			
		62.5	125	250	500
Carrot					
G-negative bacteria	*Salmonella enterica*	57.03 ± 2.42 ^b^	44.37 ± 1.14 ^c^	35.40 ± 2.18 ^c^	23.19 ± 1.63 ^c^
	*Serratia marcescens*	56.64 ± 2.19 ^b^	45.85 ± 2.78 ^c^	33.60 ± 2.11 ^cd^	23.16 ± 1.57 ^c^
	*Shigella sonnei*	57.13 ± 2.10 ^b^	45.51 ± 2.89 ^c^	33.46 ± 2.20 ^cd^	24.92 ± 2.78 ^c^
	*Yersinia enterocolitica*	56.40 ± 2.56 ^b^	43.52 ± 2.22 ^c^	35.66 ± 2.12 ^c^	24.40 ± 2.07 ^c^
G-positive bacteria	*Listeria ivanovii*	86.21 ± 2.15 ^a^	75.94 ± 4.33 ^a^	64.07 ± 2.68 ^a^	56.45 ± 1.25 ^a^
	*Listeria monocytogenes*	84.23 ± 2.29 ^a^	63.33 ± 2.20 ^b^	55.90 ± 3.98 ^b^	45.26 ± 4.31 ^b^
	*Staphylococcus aureus*	86.26 ± 1.22 ^a^	75.12 ± 0.67 ^a^	66.43 ± 1.06 ^a^	56.54 ± 1.46 ^a^
	*Streptococcus pneumoniae*	87.39 ± 1.73 ^a^	75.98 ± 4.07 ^a^	66.40 ± 2.56 ^a^	55.70 ± 3.78 ^a^
Yeast	*Candida albicans*	44.63 ± 1.86 ^c^	34.60 ± 0.06 ^d^	26.40 ± 2.63 ^de^	−14.85 ± 2.67 ^e^
	*Candida glabrata*	47.43 ± 1.59 ^c^	35.00 ± 2.56 ^d^	24.70 ± 3.10 ^e^	−15.96 ± 3.29 ^e^
	*Candida krusei*	45.74 ± 2.01 ^c^	33.83 ± 2.29 ^d^	24.22 ± 3.33 ^e^	−14.92 ± 2.78 ^e^
	*Candida parapsilosis*	44.88 ± 2.56 ^c^	35.43 ± 2.88 ^d^	24.29 ± 3.31 ^e^	−14.26 ± 1.65 ^e^
	*Candida tropicalis*	45.74 ± 2.00 ^c^	35.74 ± 1.01 ^d^	27.29 ± 2.57 ^de^	−16.40 ± 1.69 ^e^
BFB	*Salmonella enterica*	57.50 ± 0.65 ^b^	44.34 ± 1.15 ^c^	23.49 ± 1.11 ^e^	14.59 ± 2.88 ^d^
Potato					
G-negative bacteria	*Salmonella enterica*	45.96 ± 2.89 ^b^	55.70 ± 2.87 ^b^	66.77 ± 2.93 ^b^	86.56 ± 2.73 ^ab^
	*Serratia marcescens*	46.47 ± 2.73 ^b^	56.82 ± 1.06 ^b^	66.77 ± 2.93 ^b^	84.79 ± 3.39 ^b^
	*Shigella sonnei*	46.32 ± 2.68 ^b^	57.06 ± 1.41 ^b^	64.18 ± 2.30 ^b^	85.52 ± 1.85 ^b^
	*Yersinia enterocolitica*	45.60 ± 2.31 ^b^	58.24 ± 0.63 ^b^	66.36 ± 2.69 ^b^	84.51 ± 2.76 ^b^
G-positive bacteria	*Listeria ivanovii*	87.29 ± 3.36 ^a^	75.29 ± 3.38 ^a^	66.73 ± 2.90 ^b^	56.59 ± 2.16 ^c^
	*Listeria monocytogenes*	87.32 ± 2.62 ^a^	77.95 ± 1.40 ^a^	66.47 ± 2.24 ^b^	55.42 ± 3.15 ^c^
	*Staphylococcus aureus*	87.32 ± 2.62 ^a^	76.42 ± 2.78 ^a^	65.30 ± 2.31 ^b^	55.70 ± 2.87 ^c^
	*Streptococcus pneumoniae*	86.71 ± 2.11 ^a^	77.66 ± 2.70 ^a^	66.70 ± 1.23 ^b^	56.85 ± 2.56 ^c^
Yeast	*Candida albicans*	26.54 ± 3.23 ^d^	33.74 ± 2.60 ^c^	45.73 ± 2.55 ^c^	54.33 ± 2.90 ^c^
	*Candida glabrata*	25.00 ± 3.27 ^d^	32.74 ± 1.58 ^c^	46.14 ± 2.64 ^c^	54.15 ± 2.72 ^c^
	*Candida krusei*	25.77 ± 3.81 ^d^	36.84 ± 2.61 ^c^	44.76 ± 3.80 ^c^	54.88 ± 1.25 ^c^
	*Candida parapsilosis*	27.81 ± 1.12 ^cd^	33.81 ± 0.69 ^c^	44.48 ± 1.98 ^c^	56.47 ± 4.34 ^c^
	*Candida tropicalis*	25.70 ± 4.16 ^d^	33.26 ± 1.54 ^c^	45.81 ± 0.91 ^c^	55.00 ± 1.64 ^c^
BFB	*Salmonella enterica*	35.44 ± 3.22 ^c^	54.93 ± 2.31 ^b^	78.43 ± 1.77 ^a^	94.15 ± 3.12 ^a^

Data are the mean (±SD) of 3 samples. Different letters in each column (for each vegetable model: carrot and potato) refer to significant differences (Tukey, *p* ≤ 0.05).

**Table 6:** Insecticidal activity of SAEO against *Megabruchidius dorsalis* (*n* = 50).

SAEO Concetration (%)	Quantity of Living Insects	Quantity of Dead Insects	Repellent Activity (%)
100	0	100	100.00 ± 0.00
50	10	90	90.00 ± 0.00
25	40	60	60.00 ± 0.00
12.5	60	40	40.00 ± 0.00
6.25	90	10	10.00 ± 0.00
3.125	100	0	0.00 ± 0.00
Control group	100	0	0.00 ± 0.00

## Data Availability

The original contributions presented in the study are included in the article; further inquiries can be directed to the corresponding author.
